# Incidence of acute respiratory infections in preschool children in an outpatient setting before and during Covid-19 pandemic in Lombardy Region, Italy

**DOI:** 10.1186/s13052-022-01221-w

**Published:** 2022-02-03

**Authors:** Chiara Mameli, Marina Picca, Roberto Buzzetti, Maria Elisabetta Pace, Raffaele Badolato, Claudio Cravidi, Gian Vincenzo Zuccotti, Paola Marchisio, for the Italian Society of Paediatrics Lombardy Section, Marco Sala, Maria Elisabetta Di Cosimo

**Affiliations:** 1 Department of Pediatrics, V Buzzi Children’s Hospital, Milan, Italy; 2 Department of Biomedical and Clinical Science L.Sacco, Università di Milano, Milan, Italy; 3 Italian Primary Care Paediatrics Society (SICuPP), Lombardy, ATS Milano, Milan, Italy; 4 Clinical epidemiologist, Ranica, Bergamo Italy; 5 Fondazione IRCCS Ca’ Granda Ospedale Maggiore Policlinico, Pediatric Highly Intensive Care Unit, Milan, Italy; 6 Department of Pediatrics, Università di Brescia, “Istituto di Medicina Molecolare Angelo Nocivelli”, ASST Spedali civili, Brescia, Italy; 7 ATS Pavia, Pavia, Italy; 8 Università di Milano, Milan, Italy

**Keywords:** respiratory infection, children, COVID-19

## Abstract

**Introduction:**

The incidence of acute respiratory tract infections (ARTIs) in children is difficult to estimate because they are typically treated in outpatient settings and the majority of epidemiological data originate from hospital settings and refer to the most severe illnesses. Therefore, the incidence of ARTIs in a real-world setting remains largely unexplored. Therefore, this study aims to estimate the incidence of ARTIs, upper respiratory tract infections (URTIs), and lower respiratory tract infections (LRTIs) in children aged 0–5 years in an outpatient setting.

**Methods:**

This prospective cohort study was conducted in Lombardy, Italy, from October 1st, 2019, to March 31st, 2021, before and during the COVID-19 pandemic that began in March 2020. Caucasian healthy children aged 0–5 years were recruited from 69 Family Pediatricians (FP) and followed-up in an outpatient setting. Data were collected whenever a child was referred to FP and ARTI was diagnosed (Covid-19 related ARTI were excluded)**.** The primary outcome was an estimate of the incidence of ARTIs. The incidence of ARTIs in different age groups and the effect of the COVID-19 pandemic on the incidence of ARTIs were secondary outcomes.

**Results:**

We enrolled 484 children**,** 249 male (51.8%), mean age of 2.39 ± 1.68 years. The mean estimated incidence of ARTIs was 12.1/100 children × 30 days (95% CIs: 9.5–12.9), with the highest value observed in infants aged 1–12 months (24.9/100 children × 30 days; 95% CIs: 17.6–28.9). The mean estimated incidence of URTIs was higher than that of LRTIs (8.3 – CIs: 7.6–8.9 vs 3.8/100 children × 30 days – CIs: 6.4–4.3, respectively). The comparison of ARTIs, which occurred in the pre-pandemic winter, to those measured during the COVID-19 pandemic, revealed an impressive 82.1% drop in the incidence rate (CIs: 77.8–85.7).

**Conclusions:**

This study showed that infants aged 1–12 months are more likely to develop ARTIs than older children and that COVID-19 pandemic has dramatically altered the epidemiology of ARTIs in children aged 0–5 years.

**Supplementary Information:**

The online version contains supplementary material available at 10.1186/s13052-022-01221-w.

## Introduction

Acute respiratory tract infections (ARTIs) are the most common type of childhood disease [1]. Each year, these infections impose an enormous burden on the healthcare system (frequent medical consultations, hospitalizations, and antibiotic prescriptions) and on society in high income countries (parental absenteeism and loss of productivity) [1]. ARTIs, especially those involving the upper airways, are particularly common in preschool children [2]. Their incidence is often difficult to estimate because ARTIs are typically treated in outpatient settings and the majority of available epidemiological data are collected in hospital settings and are referred to as the most severe respiratory illness [3–6].

Given the paucity of evidence, the burden of respiratory infections in a real-world setting remains unclear, largely unexplored in preschool children, and appears to be an intriguing field for an observational study. As a result, we conducted a longitudinal cohort study in September 2019, which aimed to describe the epidemiology of ARTIs in a cohort of preschool children in the Lombardy region (Italy) followed in an outpatient setting. After 6 months, in March 2020, Lombardy experienced one of the first and deadliest COVID-19 outbreaks in the world, forcing the Health Authorities to lock down the entire country, completely close all schools, and suspend all work and sports activities from March 9 to May 3, 2020 [7]. In high COVID-19 incidence area (the so called “red zone”) such as Lombardy schools remained closed until June 2020. Schools then reopened for the new scholastic year in September 2020 and remained open until March 2021, when schools of all grades were closed again to contain the spread of the SARS-coV-2 following the onset of the third pandemic wave [8]. The dramatic COVID-19 outbreak and the widespread adherence to social distancing, hand washing, wearing of face masks, and lockdown have undoubtedly had an impact on the epidemiology of ARTIs in children. According to reports from different parts of the world, ARTIs in children decreased during the pandemic as a result of the combination of the different measures adopted to combat the pandemic [9–14].

In this “unique scenario”, the purpose of our prospective cohort study was to estimate the incidence of ARTIs in preschool children aged 0–5 years, who were monitored in an outpatient setting from October 2019 to March 2021, before and during the COVID-19 pandemic in Italy’s Lombardy Region.

## Methods

### Study population

This prospective cohort study was performed on a convenience sample of 69 family pediatricians (FPs) working for the Italian National Health System in Lombardy, which provides free medical care to all children in Italy. The FPs were enrolled by the Italian Primary Care Pediatrics Society (SicuPP) and the members of the Italian Pediatric Society (Lombardy Section). All FPs joined the study as volunteers.

The recruitment of FPs took place from September 1 to September 20, 2019. At the end of September 2019, all the recruited FPs attended a meeting to discuss the study procedure. Each FP was requested to recruit up to 10 children aged 0–5 years between October 2019 and January 2020. Healthy Caucasian preschool children of both sexes from 1 month to 5 years were eligible for the study. Children with conditions or diseases predisposing them to recurrent respiratory infections such as prematurity (gestational age < 37 weeks), congenital abnormalities of the respiratory tract, congenital or acquired immunodeficiency including cystic fibrosis, cardiovascular, renal, and hematological diseases, and Down syndrome were excluded.

For the purposes of the study, the recruited population was divided into 6 groups (children aged 0, 1, 2, 3, 4, and 5 years) according to age. Infants were defined as children aged between 1 and 12 months.

### Study protocol

At recruitment, each FP completed a case report form (CRF) which included socio-demographic information about each child. Parents of enrolled children were instructed to contact the FP if their child developed a fever (defined as an axillary temperature > 38 °C) or became ill [15]. The FP carried out a full clinical examination and, if an ARTI was diagnosed, a CRF was completed with the specific diagnosis in accordance with the international and statistical classification of diseases and related health problems [16]. Based on the site infection, a common cold, tonsillitis, pharyngitis, and otitis media were considered upper respiratory tract infections (URTIs), whereas infections below the epiglottis such as laryngitis, bronchiolitis, acute bronchitis, and pneumonia were considered lower respiratory tract infection (LRTIs) [17, 18]. The diagnostic criteria for each disease were discussed in an in-presence meeting and agreed upon by FPs prior to enrolling the first child.

Upon enrollment, FPs were instructed to perform follow-up phone calls to parents every 15 days to remind them of the study procedures and to monitor participants’ adherence to the protocol. Each child was followed from the day of recruitment up to March 30, 2021 (end of the study). The flow of patients during the study is described in the Results Section.

A single episode was taken into consideration if at least 2 medical visits within 14 days reported the same ARTIs diagnosis during continuous respiratory symptoms.

For this analysis, the pre-pandemic period of COVID-19 was defined as October 1st, 2019, up to February 28th, 2020, and the pandemic period as March 1, 2020 up to March 30th, 2021 (end of the study), based on national epidemiological data characterizing COVID-19 as a pandemic [19]. Italy was placed on a nationwide lockdown from March 9th, to May 3rd, 2020.

The winter period is defined as November to February.

During the COVID-19 pandemic, children with possible COVID-19 signs and symptoms were swabbed for SARS-COV-2 infection, as recommended by the National Health Authorities [20]. If the child was tested negative for COVID-19 and had symptoms of ARTIs the full clinical examination was performed and CRF was completed, and the diagnosis was recorded. Confirmation of COVID-19 was defined as the detection of SARS-CoV-2 in nasopharyngeal samples through RT–PCR.

The study was conducted in accordance with the Declaration of Helsinki and was approved in September 2019 by the Ethical Committee of the Fondazione IRCCS Ca′ Granda Ospedale Maggiore Policlinico, Milan, Italy (752–2019). Each child’s parents gave their written consent. Physicians did not receive any payment incentives for the study. The study procedures were free for all patients.

### Outcomes

The primary outcome was the estimation of the incidence of ARTIs in children aged 0–5 years (see “Statistical analysis” for further details). The secondary outcomes were the estimation of the incidence of ARTIs in different age groups, and the effect of the COVID-19 pandemic on these estimates.

### Statistical analysis

The sample size was calculated as follows. By recruiting 70 children for each of the 6 annual age groups (420 children in total) and for each of the strata identified for each month and each age group, we expected an average of 2100 children x days of attendance. Assuming that 5 to 50% of the children are afflicted by ARTIs across the strata, we estimated an incidence of 5 cases/100 children × 30 days (95% CI: 1.2–13.6) to 50 cases/100 children × 30 days (95% CI: 34.8–69.5) within each stratum (3).

The descriptive analysis of the sample of recruited children was carried out using the following criteria: a) for the numerical variables, the median value and the interquartile range, that is, the difference between the values of the third and first quartile; and b) for categorical variables, the absolute and relative frequencies for each of their values. The incidence rate was calculated as the number of events observed (episodes reported by pediatricians for each of the age groups at the time of the episode, in completed years, and for each of the months analyzed), divided by the time at risk of event for each observation period. Each child could experience more than one episode in the same calendar month. The ratios obtained in this manner were normalized as the number of episodes reported per 100 children × 30 days, and this analysis was repeated for episodes of infection affecting both the upper and lower respiratory tracts. The Poisson distribution was used to calculate the 95% confidence intervals (CIs) for incidence rates [21, 22]. The weighted average of the incidence rate in the two winter periods (November, December, January, and February of the year 2019–20 versus the same period the following year 2020–21) were then calculated, as well as the 95% confidence intervals for incidence rate differences [23].

## Results

**Fig. 1:**
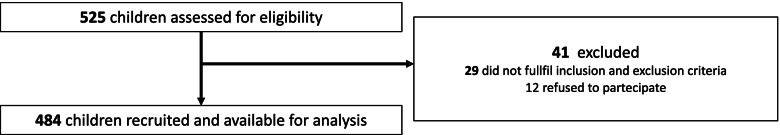
Flow of patients during the study

Five hundred twenty-five children were assessed for eligibility between October 1st, 2019 and January 30th, 2020. 29 children did not meet the inclusion and exclusion criteria, and 10 refused to participate; hence, we enrolled 484 children with a mean age of 2.39 ± and a standard deviation (SD) of 1.68 years (Fig. 1). The flow of children during the follow-up period is reported in Additional file 1.

249 (51.8%) of the recruited children were male and 340 (72.8%) were born through vaginal delivery. Children who were exclusively breastfed make up 43.5% of the sample (*n* = 462), with a median duration of 6 months of breastfeeding. At the time of recruitment, almost all the cohort (*n* = 438; 97.8%) had been administered at least 1 dose of hexavalent vaccine and at least one dose of pneumococcal vaccine (*n* = 448; 91.5%). The vast majority of the children (*n* = 404; 85.8%) attended kindergarten (IQR 1.91). The mean age of enrollment into kindergarten was 1.89 ± 1.31 SD, while the median daily attendance was of 7 h/day (IQR 1.8). The majority of children (97.2%) eat lunch in the community, while about half (58.1%) remain for an afternoon rest. 61.8% of the children use or have used pacifiers in the past. The children’s demographic characteristics are reported in Table 1.

**Table 1:** Characteristics of the study subjects

	CHILDREN	
	N	%
Male gender (***n*** = 484)	249	51.8
**Age group**		
0–1 years	131	27.1
1–2 years	111	22.9
2–3 years	70	14.5
3–4 years	62	12.8
4–5 years	61	12.6
5–6 years	49	10.1
**Mode of delivery (*n*** **= 467)**		
Vaginal	340	72.8
Caesarean	127	27.2
**Type of feeding (n = 462)**		
Excusive breastfeeding	201	43.5
Exclusive Formula	83	18.0
Mixed	178	38.5
**Vaccination** ^**a**^		
Hexavalent (*n* = 448)	438	97.8
Pneumococcal conjugate (*n* = 448)	410	91.5
MMR (*n* = 450)	323	71.8
Meningococcal B (*n* = 448)	336	75.0
Meningococcal C (*n* = 449)	324	72.2
Rotavirus (*n* = 448)	211	47.1
Influenza (2020–2021 season) (*n* = 248)	151	60.9
Influenza (past seasons before 2020) (*n* = 463)	77	16.6
**Allergies** (***n*** **= 470)**		
Inhalant allergy	8	1.9
**Indirect smoke (*n*** **= 472)**		
Yes	169	35.8
No	303	64.2
**Day care attendance (*n*** **= 471)**		
Yes	404	85.8
No	67	14.2
**Meal at Kindergartend (*n*** **= 355)**		
Yes	345	97.2
No	10	2.8
**Afternoon nap at Kindergartend (*n*** **= 353)**		
Yes	205	58.1
No	148	41.9
**Use of pacifiers (*n*** **= 456)**		
Yes	282	61.8
No	174	38.2
**Siblings (*n*** **= 468)**		
0	161	34.4
1	252	53.8
≥ 2	55	11.8

*MMR* measles, mumps and rubella vaccine. ^a^ At least 1 dose of vaccine

Table 2 illustrates the incidence estimates (cases/100 children × 30 days) and the 95% CIs of ARTIs in children by age and calendar month during the study period. The estimated mean incidence of ARTIs in children aged 0–5 years was 12.1/100 children × 30 days (95% CIs: 9.5–12.9). The estimated incidence of ARTIs was the highest in infants (24.9/100 children × 30 days; CI:s 17.6–28.9) over the entire observation period. During the COVID-19 pandemic, incidence estimates reached the lowest value in July 2020, when no episodes of ARTIs were reported in the entire cohort of children.

**Table 2:** Estimates of Incidence (cases/100 children/30 days) of acute respiratory tract infections (ARTIs) in children by age and calendar months

	Oct 19	Nov 19	Dec 19	Jan 20	Feb 20	Mar 20	Apr 20	May 20	Jun 20	July 20	Aug 20	Sept 20	Oct 20	Nov 20	Dec 20	Jan 21	Feb 21	Mar 21	Mean	95° CIs	
	Pre pandemic months					COVID-19 Pandemic months															
**0 ys**	69,8	29,7	33,1	45,2	37,0	15,0	1,7	0,0	0,0	0,0	0,0	9,3	18,6	0,0	0,0	0,0	0,0	0,0	24,9	17,6-28,9	
**1 ys**	96,6	50,9	58,4	42,0	21,7	5,6	0,0	2,0	0,7	0,0	1,3	6,4	8,7	11,9	7,9	2,0	10,4	4,3	13,1	9,9-14,8	
**2 ys**	77,9	60,0	46,4	25,1	26,2	2,5	1,3	0,0	1,2	0,0	1,1	4,2	8,6	10,7	5,2	1,6	11,8	2,3	10,5	7,2-12,3	
**3 ys**	99,7	43,0	35,2	36,8	35,4	1,4	0,0	0,0	0,0	0,0	4,3	9,3	5,9	1,5	1,4	1,4	10,4	2,7	11,2	7,5-13,3	
**4 ys**	81,6	43,6	17,8	15,5	19,4	1,7	0,0	0,0	0,0	0,0	1,7	8,4	10,9	6,5	4,8	3,1	4,9	0,0	8,4	5,8-10,4	
**5 ys**	61,2	26,7	25,1	29,6	25,8	5,0	1,7	1,6	0,0	0,0	0,0	3,4	5,2	5,2	9,6	0,0	9,3	0,0	8,1	5,3-10,1	
**All** ^**a**^	82,9	42,4	38,7	34,4	27,3	5,4	0,6	0,8	0,4	0,0	1,6	6,3	8,5	8,2	5,8	1,7	9,8	2,0	12.1	9.5–12.9	
**95°CIs**	54,7 100,6	30,8 50,5	24,6 44,9	23,2 40,1	18,9 32,5	3,2 7,9	0,1 1,9	0,2 2,2	0,1 1,6	0,0 0,8	0,6 3,2	3,8 9,1	4,9 11,7	5,1 11,4	3,3 8,6	0,6 3,4	6,2 13,5	0,8 4,0	9.5		
																			12.9		

*CIs* confidence intervals, *Ys* years; 19: year 2019; 20: year 2020; ^a^: all children

In Table 3, ARTIs are differentiated as URTIs or LRTIs based on their location, and incidence estimates are given by age and calendar months. The mean incidence estimates of URTIs were higher than those reported for LRTIs in children aged 0–5 years over the entire observation period (8.3/100 children × 30 days – CIs: 7.6–8.9 v. 3.8/100 children × 30 days – CIs: 3.4–4.3, respectively). The incidence estimates of URTIs were highest in infants (16.8/100 children/30 days; CIs: 13.9–20.2). Similarly, the incidence of LRTIs also peaked in children in their first year of life (8.1/100 children/30 days; CIs: 6.1–10.5).

**Table 3:** Estimates of Incidence (cases/100 children/30 days) of upper (panel A) and lower tract infections (panel B) children by age and calendar months

	Oct 19	Nov 19	Dec 19	Jan 20	Feb 20	Mar 20	Apr 20	May 20	Jun 20	July 20	Aug 20	Sept 20	Oct 20	Nov 20	Dec 20	Jan 21	Feb 21	Mar 21	Mean	95° CIs	
	Pre pandemic months					COVID-19 Pandemic months															
A																					
**0 ys**	46,5	23,5	18,9	26,1	27,2	13,7	1,7	0,0	0,0	0,0	0,0	9,3	18,6	0,0	0,0	0,0	0,0	0,0	**16,8**	13,9–20.2	
**1 ys**	63,3	46,5	37,0	24,5	16,3	4,9	0,0	2,0	0,7	0,0	0,7	6,4	6,5	8,8	7,0	1,0	9,1	2,9	**9,5**	8,2–10.9	
**2 ys**	51,9	37,8	17,4	17,2	12,4	2,5	1,3	0,0	1,2	0,0	1,1	4,2	4,8	9,7	4,3	1,6	9,2	2,3	**6,7**	5,5-8,1	
**3 ys**	62,9	28,7	15,1	24,5	23,1	1,4	0,0	0,0	0,0	0,0	4,3	6,2	1,5	1,5	0,0	1,4	9,0	2,7	**7,1**	5,7-8,8	
**4 ys**	56,5	34,9	8,9	9,3	15,8	1,7	0,0	0,0	0,0	0,0	1,7	6,7	9,3	4,9	3,2	3,1	4,9	0,0	**6,3**	4,8-8,0	
**5 ys**	30,6	19,1	11,4	18,5	20,3	1,7	1,7	0,0	0,0	0,0	0,0	3,4	5,2	5,2	8,0	0,0	7,5	0,0	**5,5**	4,1-7,1	
**All** ^**a**^	**53,9**	**32,5**	**20,4**	**21,0**	**19,0**	**4,6**	**0,6**	**0,6**	**0,4**	**0,0**	**1,3**	**5,6**	**6,0**	**6,7**	**4,7**	**1,4**	**8,2**	**1,8**	**8,3**	7,6-8,9	
**95°CIs**	41,8 68,5	26,4 39,4	16,3 25,1	17,1 25.5	15,2 23.,4	2,9 6,9	0,1 1,9	0,1 1,8	0,1 1,6	0,0 0,8	0,5 2,9	3,6 8,3	3,9 8,8	4,5 9,7	2,9 7,2	0,5 3,1	5,6 11,7	0,7 3,6	**7.6**		
																			**8.9**		
**B**																					
**0 ys**	23,3	6,2	14,2	19,1	9,9	1,4	0,0	0,0	0,0	0,0	0,0	0,0	0,0	0,0	0,0	0,0	0,0	0,0	**8,1**	6,1-10,5	
**1 ys**	33,3	4,5	21,4	17,4	5,4	0,7	0,0	0,0	0,0	0,0	0,7	0,0	2,2	3,2	0,9	1,0	1,3	1,4	**3,7**	2,9-4,6	
**2 ys**	26,0	22,2	29,0	7,9	13,8	0,0	0,0	0,0	0,0	0,0	0,0	0,0	3,8	1,0	0,9	0,0	2,5	0,0	**3,8**	2,9-4,9	
**3 ys**	36,7	14,3	20,1	12,3	12,3	0,0	0,0	0,0	0,0	0,0	0,0	3,1	4,4	0,0	1,4	0,0	1,5	0,0	**4,1**	3,0-5,5	
**4 ys**	25,1	8,7	8,9	6,2	3,5	0,0	0,0	0,0	0,0	0,0	0,0	1,7	1,6	1,6	1,6	0,0	0,0	0,0	**2,2**	1,4-3,3	
**5 ys**	30,6	7,6	13,7	11,1	5,5	3,4	0,0	1,6	0,0	0,0	0,0	0,0	0,0	0,0	1,6	0,0	1,9	0,0	**2,6**	1,7-3,8	
**All** ^**a**^	**29,0**	**9,8**	**18,3**	**13,4**	**8,3**	**0,8**	**0,0**	**0,2**	**0,0**	**0,0**	**0,2**	**0,7**	**2,5**	**1,4**	**1,2**	**0,2**	**1,6**	**0,3**	**3,8**	3,4-4,3	
**95°CIs**	20,3 40,1	6,6 14,1	14,5 22,8	10,3 17,1	5,9 11,4	0,2-2, 1	0,0 0,8	0,0 1,2	0,0 0,8	0,0 0,8	0,0 1,2	0,1 2,0	1,3 4,5	0,5 3,1	0,4 2,7	0,0 1,3	0,6 3,5	0,0 1,4	**3,4** **4,3**		

*CIs* confidence intervals, *Ys* years, 19 year 2019, 20 year 2020; ^a^: all children

Table 4 compares the incidence rates of ARTIs in the pre-pandemic winter (from November 2019 to February 2020) and the pandemic one (from November 2020 to February 2021). In comparison to the pre-pandemic winter months, the pandemic winter saw a relative reduction of 82.1% (− 82.1%, CIs: 77.8–85.7) in the incidence of ARTIs in children aged 0–5 years.

**Table 4:** Comparison of acute respiratory tract infections in children in prepandemic and Covid-19 pandemic winter months

	ARTIs		URTIs		LRTIs	
Age group	November 2019 to February 2020 (prepandemic winter months)	November 2020 to February 2021 pandemic winter months)	November 2019 to February 2020 (prepandemic winter months)	November 2020 to February 2021 pandemic winter months)	November 2019 to February 2020 (prepandemic winter months)	November 2020 to February 2021 pandemic winter months)
**0 ys**	36,5	–	23,7	–	12,8	–
**1 ys**	41,2	8,2	28,5	6,5	12,7	1,7
**2 ys**	37,0	7,2	19,5	6,1	17,6	1,1
**3 ys**	37,1	3,6	22,4	2,8	14,7	0,7
**4 ys**	21,7	4,8	15,1	4,0	6,6	0,8
**5 ys**	26,9	5,9	17,4	5,0	9,5	0,8
**All** ^**a**^	35,0	6,3	22,3	5,2	12,8	–
**All ages relative reduction (95°CIs)**	**–**	−82.1% (77,8–85,7)	**–**	−76,8% (−70,2-81,9)	–	−91,3% (86,1-95,0)

*ARTIs* acute respiratory tract infections, *LRTIs* lower respiratory tract infections, *URTIs* upper respiratory tract infections, *CIs* confidence intervals, *Ys* years ^a^: all children

During the study period, 64 patients were screened for SARS-CoV-2 infection; 8 (12.5%) tested positive. They recovered fully and did not required hospitalizations.

## Discussion

In this study of 484 children aged 0–5 years, who were followed for 18 months in an outpatient setting by an FP, the mean incidence of ARTIs was 12.1/100 children × 30 days over the course of the entire study period, which included both the pre-pandemic and COVID-19 pandemic periods. The incidence of ARTIs varied among the 5 age groups analyzed, reaching the highest value in infants (24.9/100 children × 30 days) and gradually decreasing up to the age of 5 years (8.1/100 children × 30 days). There is considerable variation in the reported incidence of ARTIs in healthy children, which limits comparisons between studies. Recruitment sites (emergency departments, pediatric wards, or outpatient settings), the country, the children’s ages, ethnicity, exposure to different environmental factors, seasonality, and the outcomes of interest are all factors that make the comparison difficult to perform [24–26]. We observed an age-dependent decrease in the incidence of ARTIs during the observation period, which is consistent with data reported by other authors worldwide [27]. This could be explained by the physiological postnatal maturation of both the innate and adaptive immune systems occurring during the first 6 years of a child’s life, as a result of repeated environmental exposure to microbes [28]. On analyzing infants of less than 1 year of age, which is the most affected age group in our population over the whole study period, we observed that they experienced the highest incidence estimates of ARTIs, which is consistent with what certain authors reported a few years ago [29, 30]. While we are aware that the incidence peak of ARTIs is usually seen when the child starts kindergarten, the mean age of daycare entry in our study group was 1.89 years [31, 32]. We can hypothesize on a number of potential factors influencing this result. Firstly, the role of the COVID-19 pandemic cannot be entirely ruled out. In fact, some authors have previously underlined that the pandemic had an affect on the circulation of respiratory viruses [14, 33–35]. In consequence, the shift of the incidence peak of ARTIs in younger age groups could have been the result of this new and unexpected epidemiological scenario. Secondly, the highest incidence in infants could reflect the fact that parents of younger children could be more likely to contact an FP to schedule a medical visit than parents of older children.

Not surprisingly, our study showed a higher incidence of URTIs when compared to LRTIs. When examining the incidence of ARTIs by calendar month, we observed a dramatic reduction in estimates when lockdown started in March 2020. The incidence continued to fall, reaching the nadir in July 2020, in the middle of the summer season. After the reopening of schools in September 2020, we observed an increase in ARTIs estimates which, however, never reached pre-pandemic values. This trend was similar when the incidence of URTIs and LRTIs was considered separately.

We also compared the incidence of ARTIs, URTIs, and LRTIs between the pre-pandemic winter months (from November 2019 to February 2020) and the pandemic ones (from November 2020 to February 2021). We observed a relative reduction in incidence estimates of 82.1, 70.2, and 86.1%, respectively, across all ages. The decrease in the rate of ARTIs may have primarily been caused by the reduction in social and educational activities such as school, sports and extracurricular activities, or day care centers, which are known to be major sources of respiratory infections [32]. It is worth noting that, during the pandemic period, the population of children aged 0–5 years did not wear masks, in accordance with international recommendations [36]. Therefore, the sustained decline in ARTIs when schools and educational activities reopened in September 2020 may have been influenced by hygiene measures, social distancing, and the wearing of masks by the older children, adolescents, and adult population [37]. These measures were, therefore, essential to protect children from respiratory infections during the pandemic period, when the COVID-19 vaccination campaign was just getting started (COVID-19 vaccine coverage in Lombardy was less than 5% in March 2021) [38].

## Limitations and strengths

This study has some limitations. First, the study was designed to assess the incidence of ARTIs in preschool children in mid-2019, when the COVID-19 pandemic did not yet exist. Therefore, the sudden outbreak was not anticipated when the study was conceived. Second, the incidence estimates of ARTIs could have been underestimated during the COVID-19 pandemic because of parents’ reluctance to seek medical assistance, especially during the lockdown period. However, we are confident that at least 80% of ARTIs were detected by this study given that FPs were the first doctors to be contacted in the event of respiratory diseases during the pandemic. Third, ARTIs were classified according to the site of infection and no etiological diagnosis was made. For the purposes of the study, FPs had no access to viral/bacterial diagnostic testing for URTIs and LRTIs, except during the COVID-19 pandemic when molecular tests to detect SARS-CoV-2 became mandatory in the presence of COVID-19 signs and symptoms. Fourth, not all infections were confirmed radiologically or in a laboratory, but were diagnosed according to clinical practice (for example, pneumonia was usually a clinical diagnosis because FPs did not have on-site radiologic capacity). Fifth, unfortunately we did not assess the severity of ARTIs and their outcomes (eg: drug prescription, admissions to emergency room and hospitalizations). Therefore, we cannot give any interpretation about the potential impact of the COVID-19 pandemic, direct and indirect, on the clinical severity of respiratory symptoms in children 0–5 years.

Finally, the number of FPs was relatively small when compared to the number of FPs working in Lombardy region (approximately 1000 FPs). However, we decided to involve into this study only FPs who were highly motivated and compliant to study procedure.

This study’s main strength lies in the fact that it is the largest cohort study, which unexpectedly revealed data on the incidence of ARTIs in preschool children in a real-word setting, both before and during the COVID-19 pandemic, using rigorous criteria and procedures.

## Conclusion

The aim of this study was to report the incidence of ARTIs in preschool children in an outpatient setting before and during the COVID-19 pandemic. Children in their first year of age are more likely to develop ARTIs than older children. The COVID-19 pandemic has altered the epidemiology of ARTIs in children aged 0–5 years. Further studies are needed to determine the long-term impact of COVID-19 measures on the epidemiology of ARTIs in children.

## Supplementary Information

**Additional file 1.**

## Data Availability

The full data set and other materials about this study can be obtained from the corresponding author on reasonable request.

## Abbreviations

ARTIs: acute respiratory tract infections
CI: confidence interval
FP: family pediatricians
LRTIs: lower respiratory tract infections
URTIs: upper respiratory tract infections
