# Supplementary material for: Incidence of acute respiratory infections in preschool children in an outpatient setting before and during Covid-19 pandemic in Lombardy Region, Italy
Source: Ital J Pediatr. 2022 Feb 3;48:18. doi: 10.1186/s13052-022-01221-w (PMC8812240; doi:10.1186/s13052-022-01221-w)
Supplement: Supplementary file 1 — Additional file 1. [file 13052_2022_1221_MOESM1_ESM.docx]

**Table S1. Number of patients during the study period by calendar months**

| **Age group** | **Oct**  **19** | **Nov**  **19** | **Dec**  **19** | **Jan**  **20** | **Feb**  **20** | **Mar**  **20** | **Apr**  **20** | **May**  **20** | **Jun**  **20** | **July**  **20** | **Aug**  **20** | **Sept**  **20** | **Oct 20** | **Nov**  **20** | **Dec 20** | **Jan**  **21** | **Feb 21** | **Mar**  **21** |  |  | |
| --- | --- | --- | --- | --- | --- | --- | --- | --- | --- | --- | --- | --- | --- | --- | --- | --- | --- | --- | --- | --- | --- |
|  | **Pre pandemic months** | | | | | **COVID-19 Pandemic months** | | | | | | | | | | | | |  |  | |
| **0 ys** | 4 | 57 | 99 | 102 | 89 | 76 | 64 | 53 | 45 | 34 | 23 | 16 | 3 | 0 | 0 | 0 | 0 | 0 |  |  | |
| **1 ys** | 4 | 50 | 82 | 110 | 131 | 135 | 142 | 147 | 146 | 149 | 143 | 142 | 140 | 131 | 118 | 104 | 87 | 75 |  |  | |
| **2 ys** | 3 | 34 | 59 | 70 | 74 | 79 | 77 | 78 | 79 | 82 | 87 | 93 | 99 | 100 | 105 | 114 | 124 | 130 |  |  | |
| **3 ys** | 2 | 30 | 52 | 61 | 66 | 67 | 68 | 71 | 71 | 70 | 69 | 65 | 67 | 69 | 72 | 71 | 72 | 75 |  |  | |
| **4 ys** | 2 | 26 | 46 | 58 | 62 | 58 | 56 | 54 | 56 | 55 | 57 | 58 | 61 | 60 | 61 | 60 | 64 | 66 |  |  | |
| **5 ys** | 4 | 20 | 33 | 50 | 54 | 55 | 58 | 59 | 59 | 61 | 56 | 59 | 57 | 57 | 59 | 62 | 60 | 56 |  |  | |
| **All** | **19** | **217** | **371** | **451** | **476** | **470** | **465** | **462** | **456** | **451** | **435** | **433** | **427** | **417** | **415** | **411** | **407** | **402** |  |  | |
|  |  |  |  |  |  |  |  |  |  |  |  |  |  |  |  |  |  |  |  |  |  |
|  |  |  |  |  |  |  |  |  |  |  |  |  |  |  |  |  |  |  |  |  |  |

Legend to Tabel S1. Ys: years
